# Results of genicular nerve ablation by radiofrequency in osteoarthritis-related chronic refractory knee pain

**DOI:** 10.3906/sag-1906-91

**Published:** 2020-02-13

**Authors:** Zafer Yasin KONYA*, Suna AKIN TAKMAZ, Hülya BAŞAR, Bülent BALTACI, Gülçin BABAOĞLU

**Affiliations:** 1 Anaesthesiology and Reanimation Clinic, Ankara Bilkent City Hospital, Ankara Turkey; 2 Department of Algology, Anaesthesiology and Reanimation Clinic, Ankara SUAM, Health Sciences University, Ankara Turkey; 3 Anaesthesiology and Reanimation Clinic, Ankara SUAM, Health Sciences University, Ankara Turkey; 4 Anaesthesiology and Reanimation Clinic, Algology Department, Gülhane Training and Research Hospital SUAM,Health Sciences University, Ankara Turkey

**Keywords:** Knee osteoarthritis, pain, genicular nerves, radiofrequency ablation

## Abstract

**Background/aim:**

The aim of this study was to investigate the medium- to long-term effects of radiofrequency (RF) ablation of genicular nerves for chronic refractory knee pain due to osteoarthritis (OA).

**Materials and methods:**

Forty-eight patients who underwent RF ablation of the genicular nerves were evaluated retrospectively. The visual analogue scale (VAS) score, Western Ontario and McMaster universities osteoarthritis index (WOMAC index), opioid and nonsteroidal antiinflammatory drug (NSAID) use score, quality of life score, and treatment satisfaction score were examined at 1, 3, and 6 months after the procedure.

**Results:**

The mean VAS scores were significantly lower at the 1-, 3-, and 6-month evaluations compared with the preoperative values (P < 0.001). A significant decrease was observed in the WOMAC index compared with preoperative values (P < 0.001). It was found that 66.7% of opioid users and 56.3% of NSAID users stopped using medication. No serious complications were encountered during or after the procedure.

**Conclusion:**

In chronic refractory knee pain due to OA, the application of RF ablation to the genicular nerve is an effective and safe treatment option in the medium to long term.

## 1. Introduction 

Osteoarthritis (OA) is a noninflammatory chronic degenerative disease characterized by progressive cartilage damage, osteophyte formation, and subchondral sclerosis. Knee OA is one of the most common joint diseases occurring in adults, and with increases in mean duration of life and obesity, it has become an important health problem all over the world [1,2]. The most important risk factor is age, and the prevalence of OA is as high as 40% in the population aged 70–75 years [3]. In elderly populations in particular, it is among the leading causes of pain, physical disability, and functional limitations.

As there is no curative treatment for OA, the current treatment approach is to increase quality of life via patient exercise training and pain control, decreasing physical and functional impairment and disability, and preventing the progression of the disease. Various treatment modalities that are conservative or surgical are employed [4]. Conservative treatment modalities include pharmacological methods (topical agents, simple analgesics, opioids, antidepressants, etc.), nonpharmacological approaches (training, exercise, physiotherapy, orthesis, acupuncture, etc.), or minimally invasive approaches like corticosteroid, viscosupplement, or platelet-rich plasma (PRP) injections to the intra-articular region. However, these modalities fail to treat advanced OA or stop its progression. In mild-to-moderate OA several surgical techniques have been established; in addition to conventional arthroscopic procedures, there are effective and safe surgical options. High tibial osteotomy (HTO) techniques, such as medial opening wedge HTO [5] and arthroscopic “L” medial release [6], are well-accepted procedures. Total knee arthroplasty (TKA) offers satisfactory outcomes in the treatment of chronic refractory knee pain and loss of function caused by severe knee OA. However, survival and complications are a source of concern. Despite satisfactory results, the application of this corrective surgery has been extremely low in many parts of the world. This is the result of available information and sociocultural factors; expectations regarding TKA also influence patient decisions regarding this procedure. In a recent study, Al-Mohrej et al. [7] set out to measure knowledge and attitudes related to TKA among the Saudi population and reported that there is still misinformation among the public concerning TKA, its indications, and its outcomes in Saudi Arabia. As emphasized by this study, efforts to address misinformation could lead to better patient–doctor interactions and increase patient satisfaction. This will allow more patients to benefit from this important and effective surgical treatment approach. 

In some patients, treatment cannot be continued due to side effects, while in others, despite the treatments administered, adequate and efficient pain control cannot be obtained [8]. In patients with OA-associated chronic knee pain that does not respond to conservative treatments, and among patients who do not want to undergo operations or find surgery is counter-indicated due to accompanying pathologies, the application of radiofrequency (RF) ablation to the intra-articular and periarticular regions is a novel and effective treatment option reported in the literature. Among recent studies, some report the application of this technique to the genicular nerves [9–19]. 

The knee joint is innervated by joint branches, termed genicular nerves, which originate from distal parts of the femoral, sciatic, and obturator nerves [20]. It is evident that many studies are needed to determine the efficacy and reliability of RF ablation methods applied to the genicular nerves, and development of procedural protocols and algorithms for users is also needed. The aim of the present study was to retrospectively evaluate the effect of conventional RF application on the genicular nerves for medium- to long-term pain control, functional improvement, and patient satisfaction in the treatment of chronic refractory knee pain associated with OA. 

## 2. Materials and methods 

### 2.1. Patient selection 

The present study was carried out in our department after approval was obtained from the local ethics committee. In the present study, data from 50 patients who were diagnosed with OA between January 2016 and January 2017 were evaluated retrospectively through the review of patient file records obtained via telephone or face-to-face interview. The patients were diagnosed using the criteria of the American College of Rheumatology (ACR). They had stage III or IV knee OA, as determined by physical examination and the Kellgren–Lawrence (KL) radiological scale. Moreover, because they had chronic refractory knee pain that did not respond to conservative treatments, the patients underwent diagnostic genicular nerve block, which resulted in a more than 50% decrease in pain; subsequently, the patients underwent conventional RF ablation in the genicular nerves. 

Patients with the following characteristics were excluded from the study: those with metabolic diseases like chronic rheumatism, inflammatory diseases like gout, advanced cardiac/renal disease, liver disease, uncontrolled diabetes, dementia, or psychiatric disorders; those who used antiplatelets/anticoagulants or had a coagulation disorder; and finally, those who had undergone intra-articular intervention in the last 3 months with infection at the site of intervention. In addition, one patient was excluded due to inadequate file records and another because an emergency cardiac operation was necessary during the follow-up period. Overall, 48 patients who fulfilled the criteria were included in the study. 

### 2.2. Application of the diagnostic block to the genicular nerves 

The application of the diagnostic block to the genicular nerves was performed by the same physician under operating theater conditions and standard monitoring (electrocardiogram [ECG], pulse oximeter, and noninvasive arterial pressure) as well as superficial sedoanalgesia accompanied by fluoroscopic imaging as an outpatient intervention. The details of the procedure are described below.

**Figure 1:**
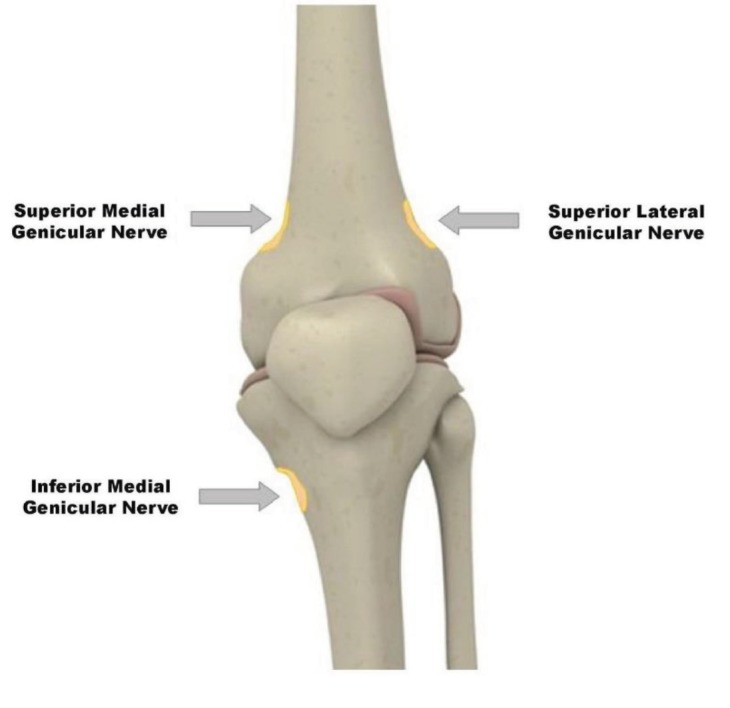
Innervation of the anterior knee joint [29].

The anatomical topography of the genicular nerves is shown in Figure 1. In the supine position with the knee at 30–40° flexion and supporting the region under the knee, the region was cleaned in accordance with the asepsis/antisepsis rules. A fluoroscope with a C arm was brought to the anteroposterior position, and the tibiofemoral joints were visualized. Subsequently, the probable positions of the superior-medial, superior-lateral, and inferior-medial genicular nerves were identified with anteroposterior images. Skin anaesthesia was provided under sterile conditions with a 25G needle and 0.5 cc of 1% lidocaine. Subsequently, each nerve was reached using 22G Whitacre spinal needles. The needles were advanced while maintaining bone contact; after their positions were corroborated in images of fluoroscopy (intersection point of 2/3 anterior and 1/3 posterior of the femur and tibia), each genicular nerve was blocked with 1 cc of 2% lidocaine. 

### 2.3. Application of conventional RF ablation to the genicular nerves

**Figure 2:**
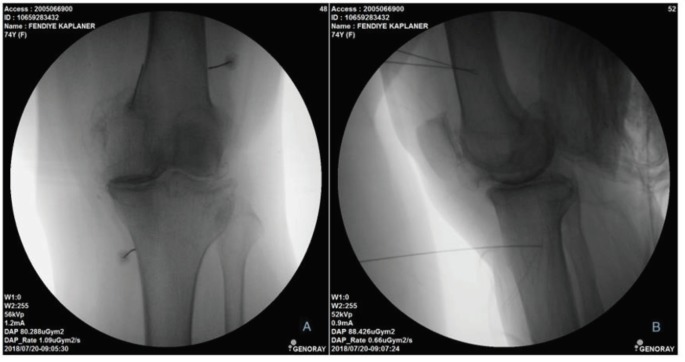
A: Anterior/posterior and B: lateral fluoroscopic images of the final electrode positions during conventional RF ablation of the genicular nerves.

Patients whose pain decreased by over 50% after diagnostic block underwent conventional RF application. The steps described in the diagnostic block were followed in a similar manner until the administration of local anaesthesia. Following local anaesthesia, 22G RF needles with active tips (100 mm) were inserted from predetermined entrance points to produce a lesion in the nerve. The needles were advanced while maintaining contact with the bone, and the positions of the needles were confirmed with lateral fluoroscopy images at the intersection point of 2/3 anterior and 1/3 posterior of the femur and tibia. Then, an electrode placed in an RF needle was connected to an RF generator (Neurotherm NT1100) and motor (0.5V), and sensorial (50 Hz) stimulations were applied to each genicular nerve to ensure the correct position of the needle. After ensuring that the motor stimulus was not received until about 2V, the RF denervation procedure was initiated. Before RF application, 2 mL of 2% lidocaine was administered to each nerve, and then conventional RF was applied at 80 °C for 60 s. The electrode position is shown in Figure 2.

### 2.4. Evaluation of the patients 

The demographic information of all the patients, as well as their clinical characteristics before the procedure and KL radiological staging, were recorded. The primary aim of the study was to determine the decrease in pain severity in the medium and long term; the secondary aim was determining the increase in medium- to long-term quality of life and patient satisfaction. For this purpose, the variables described below were used for patient evaluation at 1, 3, and 6 months and in the follow up. 

Severity of pain was evaluated with a 10 cm visual analogue scale (VAS). A VAS score <4 was considered to represent adequate analgesia. The physical functions of the knee were evaluated using the Turkish-validated form of Western Ontario and McMaster universities osteoarthritis (WOMAC) index [21]. For pain, stiffness, and physical functions, WOMAC-P, WOMAC-S, and WOMAC-PF index scores, respectively, and total WOMAC (WOMAC-T) scores obtained from the sum of these 3 parts, were recorded. For patients who were using nonsteroidal antiinflammatory drugs (NSAIDs) and/or opioids prior to the procedure, we determined whether the treatment had any effect on drug use by using a 3-point scale (1: similar, 2: decreased, 3: discontinued). To evaluate the effects of the treatment on quality of life, the following question was asked: “How did your quality of life change?” In response, the patients were instructed to choose one of four options: better, good, similar, or bad. At the end of the study, the patients were asked if they were satisfied with the treatment, and the responses were scored as follows: 1: bad, 2: moderate, 3: good, and 4: perfect.

### 2.5. Statistical analysis 

A G-Power program analysis concluded before the study calculated that, with an error margin of 0.05, statistical power of 0.95, and effect size of 0.5, at least 30 patients needed to be included in the study. To allow for missing follow-up forms, 50 patients were enrolled in the study. 

Data analysis was conducted using the SPSS 15.0 program (SPSS Inc., Chicago, IL, USA). The descriptive statistics were expressed as the mean, standard deviation, median, minimum, and maximum for continuous variables and the number of cases and percentage (%) for categorical variables. The Shapiro–Wilk test was used to evaluate whether continuous variables were normally distributed. 

The Friedman test for data that were nonlinearly distributed was used to determine whether there were statistically significant differences between the VAS, WOMAC, and quality scores at different follow-up times. When the result of the Friedman test was significant, the Wilcoxon signed-rank test was employed to determine the follow-up timepoint(s) that created the difference. When the data were linearly distributed, the follow-up timepoint creating the difference was detected with the paired-samples t-test. Whether there was significant difference in the use of opioids and NSAIDs at 1, 3, and 6 months compared with the baseline was investigated using Pearson’s chi-square test. Correlation between the VAS scores and WOMAC, satisfaction, and quality of life scores was investigated with Spearman correlation tests in data that were not linearly distributed; P <0.05 was considered significant for all results. 

## 3. Results 

**Figure 3:**
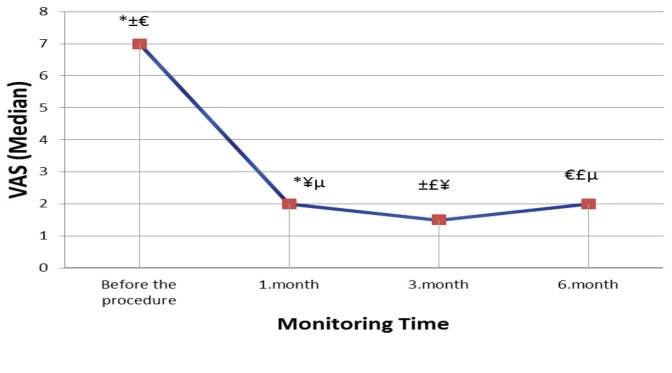
Graphic for the change in VAS scores at different follow-up times. *Difference between baseline and month 1 is statistically significant (P < 0.001). ±Difference between baseline and month 3 is statistically significant (P < 0.001). €Difference between baseline and month 6 is statistically significant (P < 0.001). ¥Difference between month 1 and month 3 is statistically significant (P < 0.001). £Difference between month 3 and month 6 is statistically sign

The demographic and clinical characteristics of the 48 patients (29 female [60.4%], 19 male [39.6%]) included in the study are listed in Table 1. The mean VAS scores of the patients included in the present study at different time points are shown in Table 2. VAS values were ˂4 in all the patients except for one patient at the end of the first month and three patients at the end of 6 months. Compared with the VAS scores recorded before the procedure, the mean VAS scores at 1, 3, and 6 months after the procedure were significantly lower (P < 0.001; Figure 3). 

**Table 1:** Demographic and clinical characteristics of the patients.

Variables	
Age (year)	77.2 ± 5.2
Sex Female Male	29 (60.4%)19 (39.6)
Weight (kg)	77.1 ± 5.1
Side Single knee Both knees	42 (87.5%)6 (12.5%)
Duration of pain (year)	4.7 ± 1.8
KL stage Stage 3 Stage 4	41 (85.4%)7 (14.6%)
Arthroscopic surgery Yes No	11 (22.9%)37 (77.1%)
Baseline opioid use Present Absent	18 (37.5%)30 (62.5%)
Baseline NSAI use Present Absent	100 (100%)0 (0%)

Data are expressed as mean ±SD (standard deviation) and n (number of patients).

**Table 2:** Mean (±SD), median, minimum, and maximum VAS scores at different follow-up times.

	n	Mean ±SD	Median	Smallest	Largest
Before the procedure * ± €	48	7.4 ± 1.3	7	5	10
1. month*	48	2.2 ± 1.0	2	0	5
3. month ±	48	1.5 ± 1.0	1.5	0	3
6. month €	48	2.0 ± 1.2	2	0	5

*The difference between the baseline and month 1 is statistically significant (P < 0.001). ±The difference between the baseline and month 3 is statistically significant (P < 0.001). € The difference between baseline and month 6 is statistically significant (P < 0.001).

**Figure 4:**
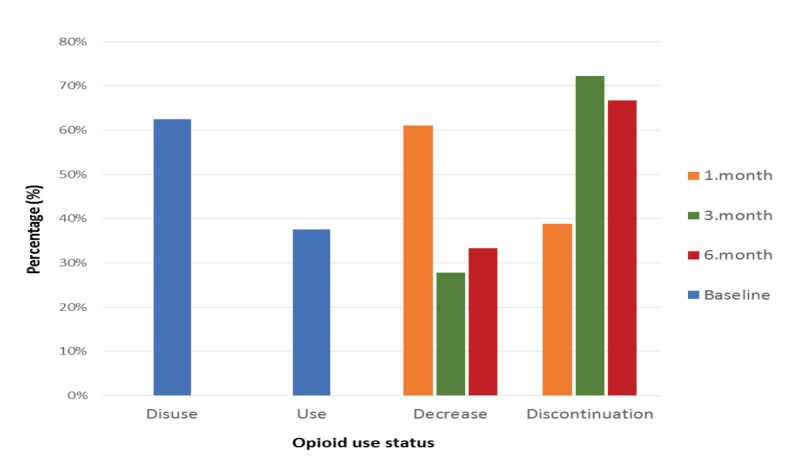
Opioid usage scores during follow-up times. Data are as expressed as the percentage of patients. *At month 1 compared to baseline, decrease in opioid use and its discontinuation is statistically significant (P < 0.001). ±At month 3 compared to baseline, decrease in opioid use and its discontinuation is statistically significant (P < 0.001). €At month 6, decrease in opioid use and its discontinuation is statistically significant (P < 0.001).

Posttreatment opioid use scores of patients who were using opioids prior to the procedure are listed in Figure 4. Prior to treatment, 18 patients (37.5%) were taking opioid analgesic drugs. At 1, 3, and 6 months after treatment, 61.1%, 27.8%, and 33.3% of the patients, respectively, had decreased their opioid use, and 38.9%, 72.2%, and 66.7% discontinued opioid use, with statistically significant changes (P < 0.001; Figure 4).

**Figure 5:**
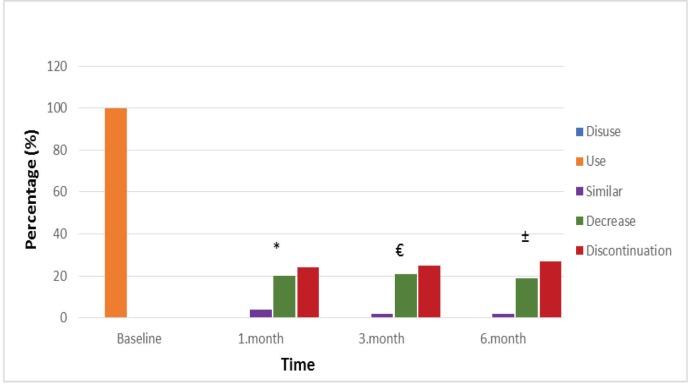
NSAID usage scores during follow-up times. *At month 1 compared to baseline, decrease in NSAID use and its discontinuation is statistically significant (P < 0.001). €At month 3 compared to baseline, decrease in NSAID use and its discontinuation is statistically significant (P < 0.001). ±At month 6 compared to baseline, decrease in NSAID use and its discontinuation is statistically significant (P < 0.001).

Before the procedure, 100% of the patients were using NSAIDs. At 1, 3, and 6 months posttreatment, 41.7%, 43.8%, and 39.6% of the patients, respectively, decreased their NSAID use, while 50%, 52.1%, and 56.3% discontinued NSAID use, with statistically significant changes between the values before the procedure and after the procedure (P < 0.001; Figure 5).

Changes in the mean WOMAC scores of the patients are shown in Table 3. Compared with the values before the procedure, the WOMAC-P, WOMAC-PF, and WOMAC-S scores decreased significantly at 1, 3, and 6 months posttreatment. Similarly, the total WOMAC scores were lower at 1, 3, and 6 months compared with the baseline, and they were significantly lower at 3 and 6 months compared with 1 month. The greatest decrease in the WOMAC-T score, when compared with pretreatment values, occurred at 6 months. 

**Table 3:** Mean WOMAC-P, WOMAC-S, WOMAC-PF, and WOMAC-T scores at different follow-up times.

	Before procedure	1. month	3.month	6.month
WOMAC-P	12.0 ± 2.3	4.9 ± 1.7*± ¥	4.1 ± 1.2*±£	3.7 ± 1.2*£¥
WOMAC-S	4.4 ± 1.4	2.7 ± 0.9*	2.7 ± 0.9*	2.7 ± 0.9*
WOMAC-PF	46.1± 6.4	28.8 ± 5.8*	28.7 ± 5.5*	28.5 ± 5.5*
WOMAC-Total	62.2 ± 9.4	36.5± 7.4*β #	35.5 ± 6.3* β	35.1 ± 6.4* #

Data are expressed as mean ±SD. *Statistically significant difference before the procedure and at 1, 3, and 6 months postprocedure (P < 0.01). ±Statistically significant difference between month 1 and month 3 (P < 0.001). £Statistically significant difference between month 3 and month 6 (P < 0.006). ¥Statistically significant difference between month 1 and month 6 (P < 0.001). βStatistically significant difference between month 1 and month 3 (P < 0.05). #Statistically significant difference between month 1 and month 6 (P < 0.001).

**Figure 6:**
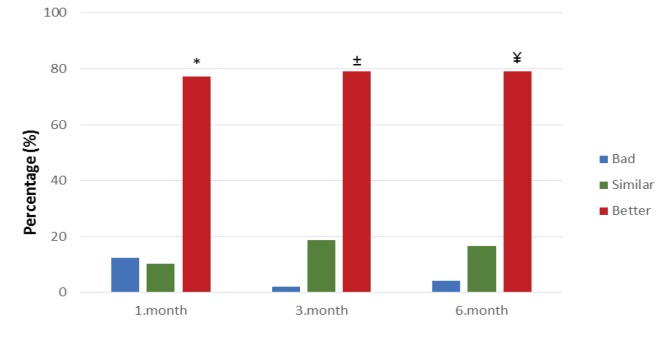
Quality of life scores during follow-up times. *Month 1 quality of life scores significantly better (P < 0.05). ±Month 3 quality of life scores significantly better (P < 0.05). ¥Month 6 quality of life scores significantly better (P < 0.05).

Significant improvement was observed in quality of life after treatment (Figure 6). At 1, 3, and 6 months after treatment, 79.1%, 79.2%, and 79.1% of the patients, respectively, reported that their quality of life was better. At 1, 3, and 6 months the quality of life scores increased as the VAS scores decreased; however, a significant correlation was only found between the 6 month VAS scores and quality of life scores (coefficient of correlation = −0.41, P = 0.003).

After treatment, patient satisfaction levels were high. The satisfaction levels were as follows: 77.1% perfect, 14.6% good, 6.3% moderate, and 2.1% bad. After the end of the study, 97.9% of the patients (all except one) reported that they would undergo the procedure again. A significant negative correlation was found between patient satisfaction and the WOMAC-T score; that is, at all timepoints, the patient satisfaction level increased as the WOMAC-T score decreased.

No serious side effects or complications occurred in association with the treatment applied. In two patients, hematoma and ecchymosis occurred at the needle sites; in one patient, painless paresthesia around the knee was evident, and this resolved spontaneously without treatment in 1 day. 

## 4. Discussion 

The present study demonstrated that, in chronic refractory knee pain associated with OA, the application of conventional RF ablation decreased analgesic use and increased quality of life and patient satisfaction. Moreover, it resulted in effective analgesia and functional improvement in the medium and long term without serious side effects. 

Since the first publication on pulsed RF application to the intra-articular region [22], studies investigating the efficacy of various RF modalities, such as conventional RF [9–16], cooled RF [17–19], and pulsed RF [23–25] applied to the intra-articular and periarticular regions in patients with chronic knee pain have appeared. Many such studies have been reported in the literature in the last decade.

The application of conventional RF to genicular nerves under fluoroscopic guidance was originally described by Choi et al. [9] in 2011. In their randomized, double-blind study comparing genicular nerve blockage and conventional RF, there was over 50% relief and associated functional improvement in knee pain for 12 weeks without any side effects. For the procedure, a 100 mm RF needle with active tip was used, and each nerve lesion was produced at 70 °C for 90 s. 

In the conventional RF application from the present study a 100 mm active-tip RF needle was used, and in a departure from Choi et al. [9], a lesion was produced at each nerve at 80 °C for 60 s. A 75% reduction in the median VAS scores was observed in the 1, 3, and 6 month measurements. When follow up was terminated at 6 months, the percentage of patients with VAS scores ≤4 was found to be quite high (93.75%). In parallel to the satisfactory analgesia obtained, analgesic consumption decreased, while quality of life increased significantly. Moreover, no serious side effects occurred. As the duration of follow up was 6 months, we could not determine how long the obtained effect lasted. However, according to observations made in clinical practice, the clinical effect was maintained for up to 1 year.

In the present study, the WOMAC OA index was also used to evaluate the efficacy of treatment, and it was compared with scores from the preprocedural period; a significant decrease was found in total and subgroup WOMAC scores at all follow-up timepoints. The results of the present study are compatible with those of many studies using WOMAC as an indicator of improvement in quality of life; similar to those studies, our results demonstrated that conventional RF considerably decreased knee pain, stiffness, and disability after 6 months of follow up [9,13,16].

In conventional RF, the size of the lesion varies depending on the distance to the nerve, duration of application, target heat, and length of the active tip. In the studies regarding conventional RF application to genicular nerves, there is no standardization in the parameters used. For example, the length of the active tip varies between 5 and 10 mm, the heat used in the procedure varies between 60–80 °C, and duration of procedure varies 90–270 s. Although comparative studies have not been carried out, it is our view that these technical differences may change the long-term of efficacy of conventional RF. For instance, in studies with 3–6 months of follow up that used 10 mm active-tip RF needles [9–12,15,16], the improvements lasted for time periods similar to those in the present study. Ikeuchi et al. [14] used a 5 mm active-tip RF needle, and the improvements disappeared within 6 months. In contrast, in a retrospective study in which the lesion was applied at 60 °C, which is generally preferred in the literature, a temperature of 80 °C achieved physical and functional improvement for 6 months, similar to the findings in other studies [12]. In the present study the lesion was produced at 80 °C using an RF needle with a 10 mm active tip, and unlike other studies, the duration of the procedure was 60 s; however, adequate physical and functional improvement was obtained. Therefore, we suggest that randomized prospective studies comparing the temperature used, duration of lesion production, as well as active-tip size, are required. 

In addition to the conventional RF application method, cooled RF, and pulsed RF methods may also be utilized. Although there has been no randomized study comparing these methods, Gupta et al. [26] evaluated the efficiency of the three methods in a review and reported that all three have similar safety and efficiency profiles. 

In the literature, there are also studies comparing RF methods with conservative treatments. One study comparing intra-articular steroid, local anaesthetic, and opioid injections with conventional RF found conventional RF application to the genicular nerves provided better analgesia and improved joint functions efficiently and reliably [16].

Radiofrequency application to genicular nerves has traditionally been carried out under fluoroscopy; now it is being carried out with ultrasonography (USG) [15,22]. Ultrasonography is easier, provides more accurate visualization of neurovascular structures, and does not involve exposure to radiation, when compared with fluoroscopy [22]. However, there is not adequate evidence at present indicating the superiority of USG to fluoroscopy; furthermore, the need for devices and the additional training required are among its disadvantages. In a recent anatomic study performed with cadavers, it was demonstrated that, although the course of the nerves was variable at the proximal area, in the distal region, where there was contact between the femur and tibia, it was constant [27]. Therefore, it is our opinion that the probability of an unsuccessful block due to anatomic variation is not high in this region, and conventional RF application to the genicular nerves can be carried out safely and efficiently under the guidance of fluoroscopy. 

In OA-associated knee pain, conventional RF application to the genicular nerves is regarded as a safe intervention with low complication rates, as no serious side effects have been reported. The results of the present study confirm this. Apart from hematoma and ecchymosis at the needle sites in two patients and painless paraesthesia lasting for 1 day around the knee that resolved spontaneously in one patient, no complications occurred. In a study by McCormick and Walega [28], an 8 mm third-degree skin burn was reported in the injection region following the application of conventional RF with a 100 mm active-tip needle at 80 °C for 90 s. Investigators have stressed that in order to prevent skin burns that may develop in RF ablation applications, especially in patients with a low body mass index, the length of the electrode active tip, degree of the lesion, and duration of the procedure should be individualized; and to avoid the risk of electrode migration, fluoroscopic images should be obtained at certain intervals. Although no complications occurred in the present study, with the increasing popularity of the procedure and emphasis in the literature on probable complications, we suggest taking precautions in accordance with the recommendations; furthermore, when necessary, treatment should be individualized. 

The retrospective nature of this study, lack of a control group, and the limited 6-month follow-up duration may be considered limitations of the present research. It is our view that this study may provide guidance for further prospective, controlled, and long-term studies comparing RF applications with both placebo and other RF methods (pulsed RF, cooled RF, etc.). 

In conclusion, conventional RF ablation application to the genicular nerves is an effective analgesia method that decreases the consumption of NSAIDs and opioids in the medium and long term, provides functional improvement with satisfactory and adequate analgesia, and increases quality of life, with high patient satisfaction and without serious side effects in chronic refractory knee pain associated with OA. However, further longer-term randomized, controlled, double-blind studies with larger patient series are required to substantiate these findings.
